# Carbamazepine, a beta-cell protecting drug, reduces type 1 diabetes incidence in NOD mice

**DOI:** 10.1038/s41598-018-23026-w

**Published:** 2018-03-15

**Authors:** Jason T. C. Lee, Iryna Shanina, Yung Ning Chu, Marc S. Horwitz, James D. Johnson

**Affiliations:** 1 Diabetes Research Group, UBC Life Sciences Institute, Department of Cellular and Physiological Sciences, Vancouver, BC Canada; 2 Department of Microbiology and Immunology, UBC Life Sciences Institute, Vancouver, BC Canada

## Abstract

Pancreatic beta-cells are selectively destroyed by the host immune system in type 1 diabetes. Thus, drugs that preserve beta-cell mass and/or function have the potential to prevent or slow the progression of this disease. We recently reported that the use-dependent sodium channel blocker, carbamazepine, protects beta-cells from inflammatory cytokines *in vitro*. Here, we tested the effects of carbamazepine treatment in female non-obese diabetic (NOD) mice by supplementing LabDiet 5053 with 0.5% w/w carbamazepine to achieve serum carbamazepine levels of 14.98 ± 3.19 µM. Remarkably, diabetes incidence over 25 weeks, as determined by fasting blood glucose, was ~50% lower in carbamazepine treated animals. Partial protection from diabetes in carbamazepine-fed NOD mice was also associated with improved glucose tolerance at 6 weeks of age, prior to the onset of diabetes in our colony. Less insulitis was detected in carbamazepine treated NOD mice at 6 weeks of age, but we did not observe differences in CD4^+^ and CD8^+^ T cell composition in the pancreatic lymph node, as well as circulating markers of inflammation. Taken together, our results demonstrate that carbamazepine reduces the development of type 1 diabetes in NOD mice by maintaining functional beta-cell mass.

## Introduction

The vast majority of type 1 diabetes clinical trials have focused on selective immune suppression and no approved therapies have been developed that directly protect beta-cells by promoting cellular health and function^1^. There is a lack of information on the molecular mechanisms of beta-cell death in type 1 diabetes, and only few examples of viable drug targets exploitable for beta-cell protection *in vivo*^2^. We recently conducted a high-throughput, multi-parameter, image-based screen to identify FDA-approved drugs from the Prestwick compound library that protect beta-cells against a cocktail of inflammatory cytokines (TNF-α, IFN-γ, IL-1β) designed to mimic type 1 diabetes conditions^3^. Carbamazepine, a use-dependent sodium channel blocker that is used clinically as an anti-epileptic, was identified as a compound that reduced beta-cell death^3^. The protective effect on beta-cells against toxic cytokines was seen with use-dependent sodium channel blockers like carbamazepine and lidocaine, but not the use-independent sodium blocker tetrodotoxin^3^. Another screen from our group found that carbamazepine promoted insulin production^4^ and other sodium channel blockers have been found to promote beta-cell function in reporter-based compound screens conducted by other groups^5^. Based on these findings, we concluded that carbamazepine merited additional study in an *in vivo* model of type 1 diabetes.

Carbamazepine blocks inward sodium currents in beta-cells and directly acts on the Nav1.7 sodium channel isoform in a use-dependent manner^3,4^. We and others have found that Nav1.7 is the principal voltage-gated sodium channel alpha subunit isoform expressed in purified beta-cells^3,6^. *Scn9a* knockout mice, which lack the Nav1.7 current, have normal insulin secretion but improved cellular insulin content with age^4,6^. We have previously shown that carbamazepine does not significantly inhibit insulin secretion from mouse islets^3^.

In the present study, we investigated the effects of carbamazepine on type 1 diabetes incidence, beta-cell function and pancreatic pathology in the non-obese diabetic (NOD) mouse model. We identified a clear difference in diabetes incidence between control and carbamazepine treated animals, with positive effects on beta-cell function. Peripheral immune cell composition and inflammation were largely unchanged in drug treated animals. Collectively, our data suggest carbamazepine protects non-obese diabetic mice from type 1 diabetes primarily via the promotion of beta-cell survival and function.

## Results

### Carbamazepine reduces diabetes incidence in NOD mice

**Figure 1:**
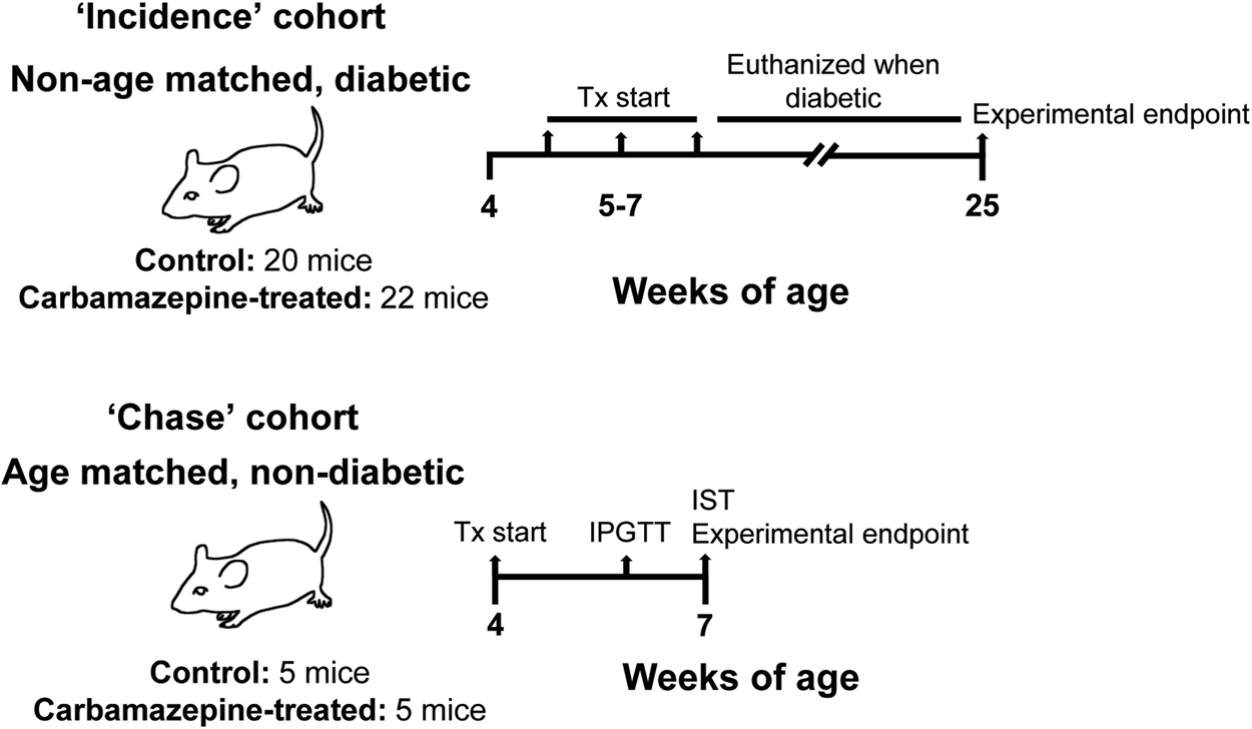
Study design. Schematic diagram of experiments conducted on non-obese diabetic mice (non-age matched – ‘incidence cohort’) or a pre-diabetic, age-matched (‘chase’ cohort).

Carbamazepine protects beta-cells against a cocktail of pro-inflammatory cytokines *in vitro*^3^. To test whether carbamazepine protects beta-cells *in vivo*, we utilized two cohorts of female, non-obese diabetic (NOD) mice: a diabetes ‘incidence’ cohort and a pre-diabetic and an age matched ‘chase’ cohort (Fig. 1). In both cohorts, mice were weaned onto diet supplemented with or without carbamazepine and assessed weekly for fasting blood glucose. There was no significant difference on food intake between the two groups. No overt changes in daily activity or behaviour were noted between the treatment groups during daily observation throughout the course of the study. One mouse treated with carbamazepine developed a mass in the front right limb after 5 weeks of treatment. Although this mass did not affect walking ability and we have no reason to believe it was related to the drug treatment, that mouse was euthanized and subsequently excluded from further analyses.

**Figure 2:**
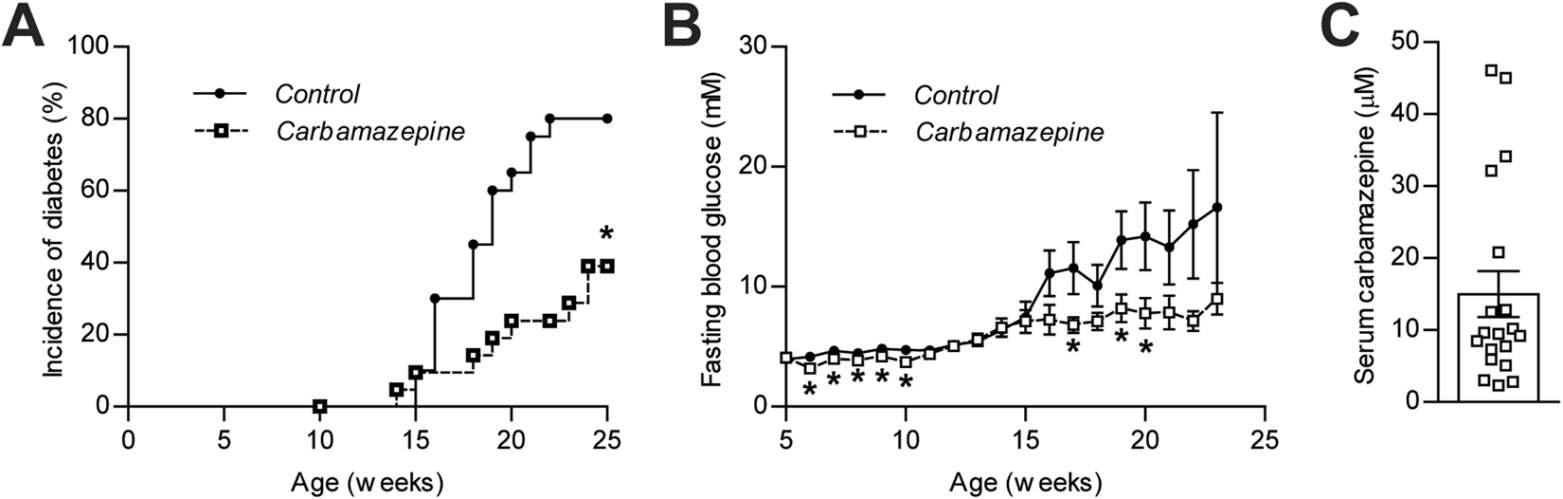
Oral carbamazepine ameliorates diabetes incidence NOD mice. Female NOD mice were weaned onto LabDiet 5053 supplemented with 0.5% w/w carbamazepine (n = 22) or LabDiet 5053 alone (n = 20). Mice were euthanized upon reaching diabetic status (2 consecutive weekly measurements of fasting blood glucose >16.0 mmol/l) and diabetes incidence was depicted via a Kaplan-Meier curve (**A**) with its corresponding fasting blood glucose (**B**). Upon euthanization, serum was collected via cardiac puncture and serum carbamazepine was quantified by ELISA (n = 19) (**C**).

We recorded that 80% of control mice in the ‘incidence’ cohort developed diabetes by the age of 25 weeks, as evidenced by at least 2 fasting blood glucose measurements above 16 mM (Fig. 2A). This is consistent with the typical diabetes incidence rate in NOD mice in our colony^7^ and in other facilities^8^. In contrast, diabetes was only observed in only 40% of the NOD mice treated with carbamazepine. Although differences in diabetes incidence diverged at about 16 weeks in age, we noted that carbamazepine-treated mice had significantly lower fasting blood glucose well prior to chronic hyperglycemia at 6–10 weeks of age (Fig. 2B). To provide an estimate of drug exposure, mean serum carbamazepine levels quantified by ELISA after euthanasia and found to range between 2 μM and 45 μM (Fig. 2C). Linear regression found no relationship between carbamazepine concentration and glycemia. Collectively, our data demonstrate that carbamazepine treatment, achieving levels used in other studies^9^, can prevent 50% of diabetes cases in our NOD mouse population within 25 weeks of age.

### Carbamazepine improves glucose tolerance in pre-diabetic NOD mice

**Figure 3:**
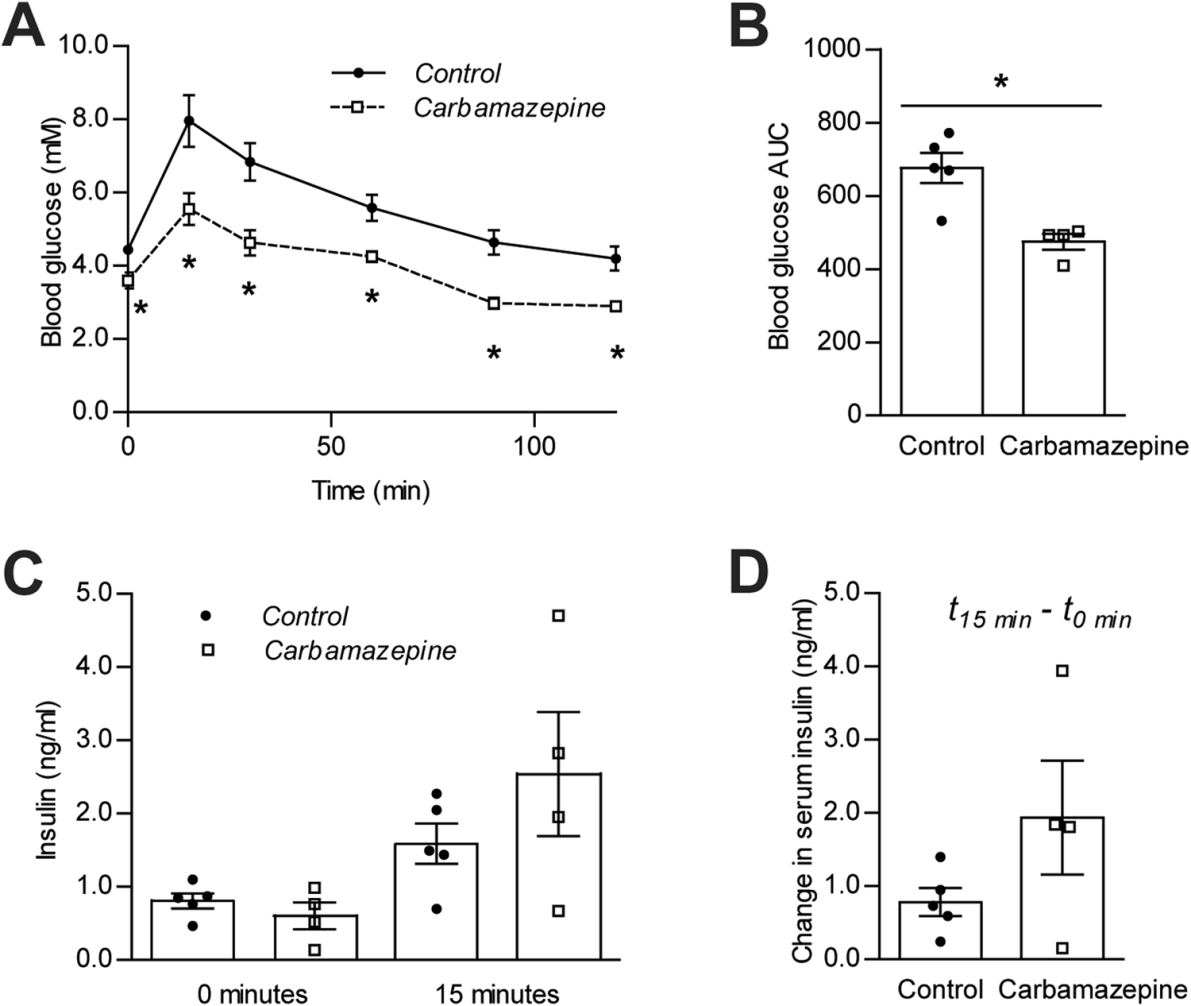
Improved glucose homeostasis in pre-diabetic NOD mice. Female NOD mice were either fed LabDiet 5053 supplemented with 0.5% w/w carbamazepine (n = 5) or LabDiet 5053 alone (n = 4). 6 week old mice were challenged with an intraperitoneal injection of D-glucose (2 g/kg) and blood glucose was assessed over a period of 2 hours (**A**) with corresponding area under the curve (AUC) quantification (**B**). One week later at 7 weeks of age, mice were challenged with 2 g/kg glucose and insulin secretion (**C**) and difference from baseline (**D**) were quantified after a 15 minute period.

In order to identify the mechanisms mediating the protection from diabetes in carbamazepine-treated NOD mice, and due to the variable age in which mice were euthanized within the ‘incidence’ cohort, we examined our ‘chase’ cohort of age-matched NOD mice sacrificed prior to the onset of hyperglycemia. Glucose tolerance at 6 weeks of age was significantly improved in carbamazepine treated mice compared to control animals (Fig. 3A and B). Glucose stimulated insulin secretion normalized to basal secretion tended to be higher in a small study including some of the same mice at 7 weeks, although the insulin measurements were highly variable (Fig. 3C and D). Collectively, these experiments suggest that beta-cell function is improved by carbamazepine rapidly after the initiation of treatment.

### Beta-cell survival following carbamazepine treatment

**Figure 4:**
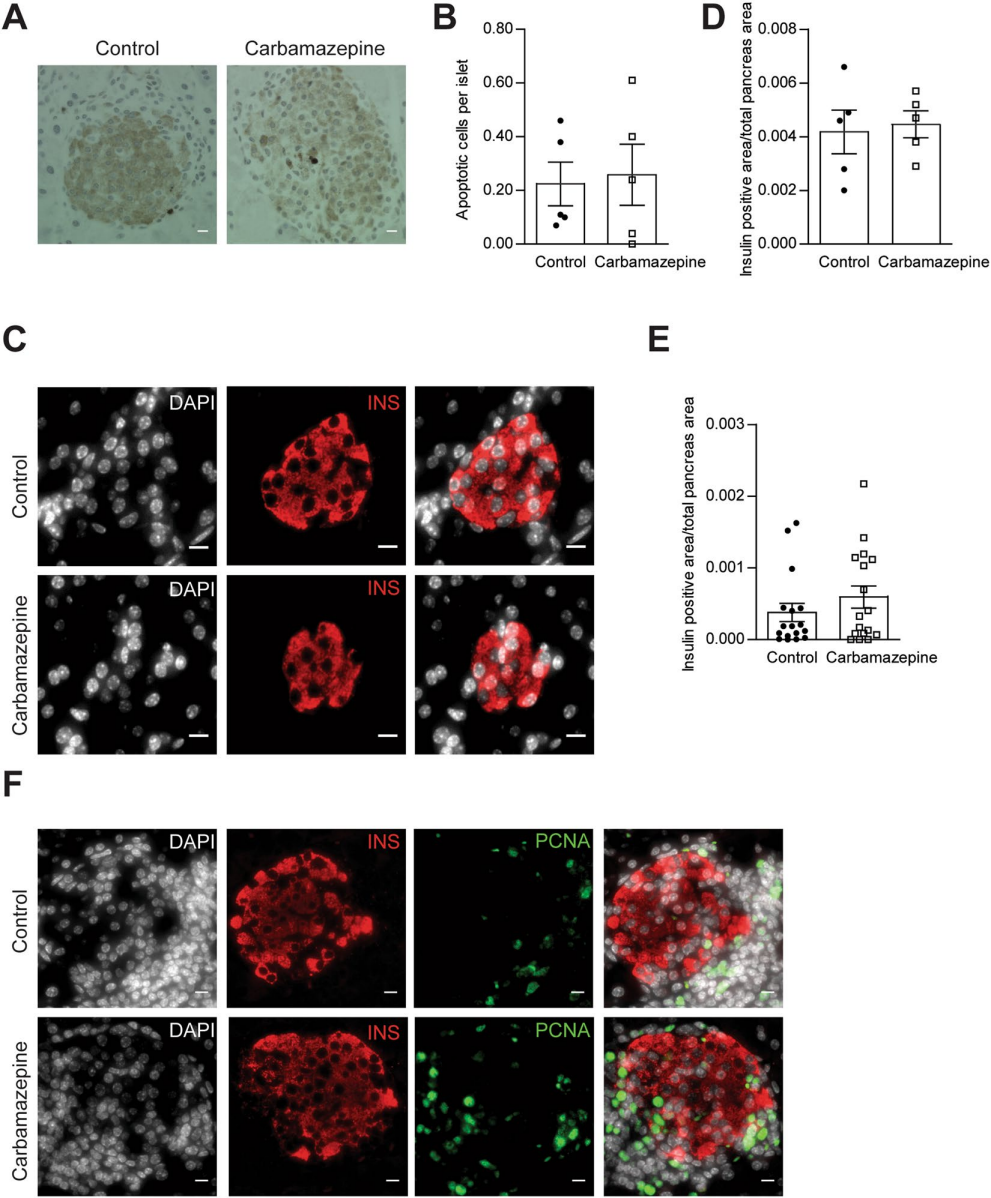
Histological assessment of beta-cell apoptosis, beta-cell proliferation and beta-cell area. Apoptotic cell death was quantified via TUNEL staining (**A**) in the pre-diabetic ‘chase’ cohort (n = 5 for control and treatment group) (**B**). Beta-cell area was quantified via insulin staining (**C**) and normalized to total pancreatic area in the pre-diabetic ‘chase’ cohort (n = 5 for control and treatment group) (**D**) and the diabetic ‘incidence’ cohort (n = 17 for control and treatment group) (**E**). Mice from the pre-diabetic ‘chase’ cohort were also stained with co-stained for PCNA and insulin (**F**). Scale bars are 10 μm.

To determine the effects of carbamazepine on beta-cell survival *in vivo* in the context of a fully competent immune system, and to test whether alterations in beta-cell mass could contribute to the improved glucose tolerance in our drug treated animals, we performed histological analyses and TUNEL staining. We found no significant differences in islet cell death as measured by TUNEL staining (Fig. 4A) in the pre-diabetic ‘chase’ cohort of mice treated with carbamazepine as compared to control (Fig. 4B), although it should be noted that this technique is notoriously insensitive due to the transient (~3 hours) nature of TUNEL labelling *in vivo* where macrophages rapidly dispose of apoptotic beta-cells^10,11^. We also tested whether physical beta-cell mass was altered in a robust way by carbamazepine treatment. We observed no statistically significant differences in beta-cell area (Fig. 4C) between treatments groups in the pre-diabetic ‘chase’ cohort (Fig. 4D) nor the ‘incidence’ cohort (Fig. 4E). Although it was noted that all mice had profound beta-cell loss with age and disease progression, it is possible that carbamazepine is efficacious in only a subset of experimental animals, consistent with Fig. 2A. While nonparametric analyses did not identify a statistical difference between control and treatment, our data hint that beta-cell preservation may potentially play a role in the protective effects of carbamazepine originally identified *in vitro* A higher powered study, and perhaps robust subgroup analysis, would be required to overcome the heterogeneity of disease incidence in the NOD model and properly test this hypothesis.

We also assessed whether carbamazepine had the ability to stimulate beta-cell proliferation *in vivo* by staining some sections for proliferating cell nuclear antigen (PCNA) (Fig. 4F). We were unable to find compelling instances of PCNA staining in insulin-positive cells. However, infiltrating mononuclear cells surrounding the islet in both treatment groups showed robust proliferation, demonstrating that the staining protocol was capable of identifying proliferating cells in the same slides. If the effects of carbamazepine were related to changes in beta-cell proliferation, they would be too subtle to detect using current approaches.

### Carbamazepine reduces insulitis in NOD mice

**Figure 5:**
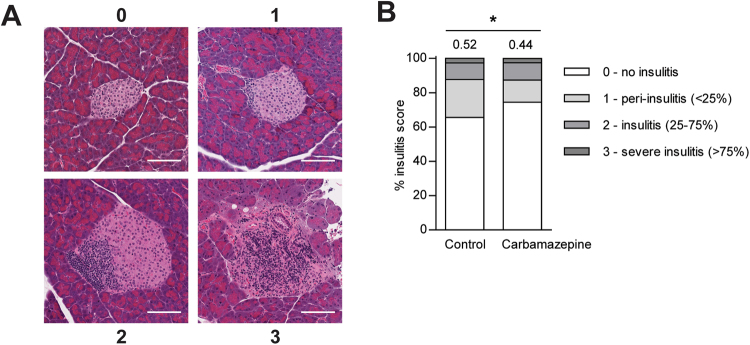
Carbamazepine treatment reduces insulitis in NOD mice. Insulitis in the carbamazepine-treated and control animals from the pre-diabetic ‘chase’ (n = 5 for control and treatment group) cohort was blindly scored in by H&E stained pancreata based on standard criteria with representative image examples shown in (**A**), and quantified with mean insulitis score (**B**). Scale bars are 100 μm.

We next assessed other markers of immune function to determine if carbamazepine had any significant immunosuppressive abilities. Immune-islet infiltration (insulitis) in H&E-stained pancreatic sections was quantified and scored according to standard criteria^7^ (Fig. 5A). At 7 weeks of age, carbamazepine treated NOD mice had an overall decrease in insulitis as compared with age-matched controls. In particular, we counted more islets with no insulitis and fewer islets with peri-insulitis with drug treatment (Fig. 5B). These results indicate that carbamazepine may, directly or indirectly, modulate the immune cell attraction to islets or infiltration into islets, perhaps by reducing β-cell immunogenicity.

### T cell populations and circulating inflammation in carbamazepine treated and control mice

**Figure 6:**
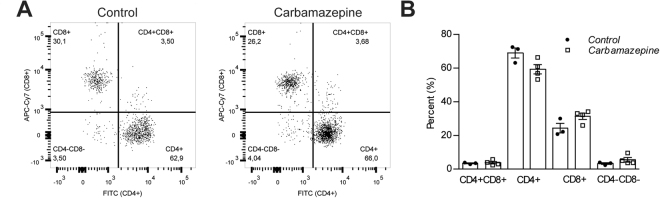
Analysis of pancreatic lymph node immune cell populations in carbamazepine-treated and untreated NOD mice. Mice from the pre-diabetic ‘chase’ (n = 3 for control, n = 4 for treatment group) cohort were euthanized and lymphocytes were isolated from the pancreatic lymph node. Cells were subjected to surface staining with CD3, CD4, and CD8 antibodies. CD3^+^ cells were gated and proportions of CD4^+^/CD8^+^ cells were analyzed (**A**) and depicted in a bar graph (**B**).

We next sought to perform experiments to assess peripheral T cell populations and systemic inflammation in animals treated with or without carbamazepine. We isolated lymphocytes from pancreatic lymph nodes from our ‘chase’ cohort and stained for markers of T cell identity. Flow cytometric analyses for CD3^+^ cells revealed no significant differences in proportions of CD3^+^CD4^+^ or CD3^+^CD8^+^ T cells (Fig. 6A and B).

**Figure 7:**
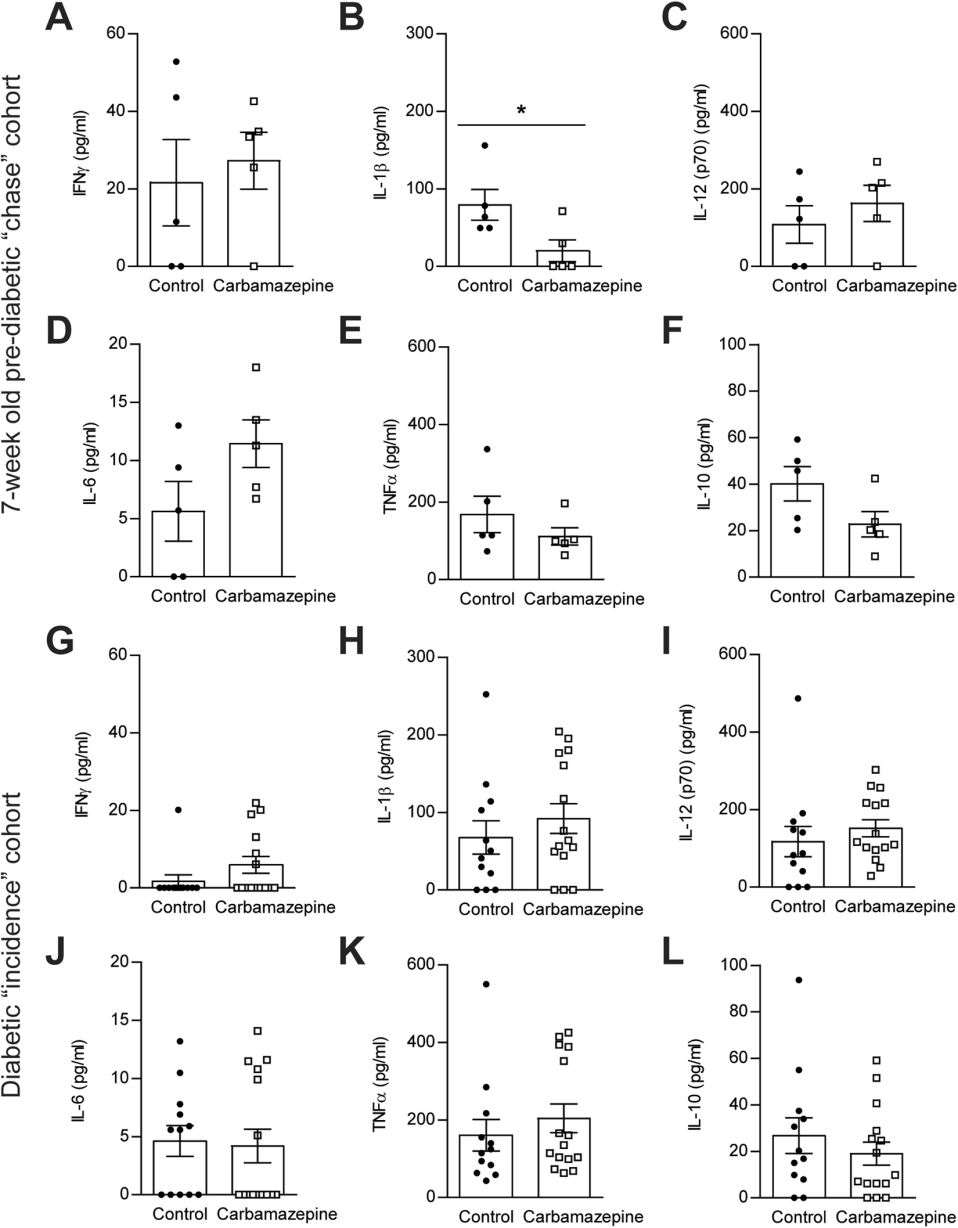
Circulating markers of inflammation in carbamazepine- treated and untreated NOD mice. Serum collected at experimental endpoint was assessed via a 7-plex mouse Th1 inflammatory Luminex kit for the pre-diabetic ‘chase’ cohort (n = 5 for control and treatment group) (**A**–**F**) and a subset of the ‘incidence’ cohort (n = 12 for control, n = 15 for treatment group) (**G**–**L**).

We also examined a panel of seven cytokines present in serum of the ‘chase’ (Fig. 7A) and ‘incidence’ cohorts (Fig. 7B) as a measure of whole animal inflammatory status. Of the seven analytes we examined, we were unable to detect IL-2 within the standard range of the assay and we therefore excluded it from further analyses. Pre-diabetic, carbamazepine treated mice (‘chase’ cohort) had significantly lower circulating IL-1β as compared to controls, but this difference was not seen in older ‘incidence’ cohort. There were no differences in circulating concentrations of IFNγ, IL-12, IL-6, TNF-α, or IL-10 in either the 7-week pre-diabetic cohort or in the incidence cohort studied up to 25 weeks of age, although many of the data distributions were bimodal in the old mice, as would be expected given the heterogeneity of disease incidence (Fig. 7A and B).

## Discussion

We used the NOD mouse model to assess the effects of a clinically relevant dose of carbamazepine on type 1 diabetes incidence. As we hypothesized based our previous *in vitro* screens and follow-up experiments in which we observed cellular-level protection and improved beta-cell function^3,4^, we found a significant decrease of diabetes incidence in mice fed a diet that included carbamazepine. We observed improved glucose tolerance in carbamazepine treated animals prior to the onset the first case of diabetes, suggesting that key cellular target of this drug may be the pancreatic beta-cell. Although our *in vitro* studies and the relatively restricted tissue expression of *Scn9a* point to the beta-cells as a likely target, it is theoretically possible that carbamazepine may affect glucose tolerance by some other mechanism.

We found early improvement in beta-cell function in NOD mice, but were unable to clearly demonstrate a robust difference in *in vivo* beta-cell survival or beta-cell area between the control and treatment groups, although the bimodal distribution of the data within the incidence cohort confounded this analysis. It is also possible that the dose of carbamazepine achieved *in vivo* was not sufficient for anti-apoptotic effects in the face of full autoimmunity. It should be noted that, 10 μM carbamazepine was not completely protective in our *in vitro* via high throughput screen^3^. By comparison, carbamazepine increased insulin mRNA and inhibited the Nav1.7 sodium channel at concentrations as low as 0.01 μm^4^. Future *in vivo* studies at multiple carbamazepine doses could provide useful information. Since neither acute carbamazepine treatment nor Nav1.7 gene deletion significantly increase insulin secretion from rodent islets directly^3,6,12,13^, it is possible that the dampening effect of this sodium channel inhibitor might be beneficial to beta-cell health.

Carbamazepine is clinically used as an anti-epileptic and is believed to act via the use-dependent inhibition of voltage-gated sodium channels^14^. Carbamazepine has additional effects that may or may not be related to its targeting of plasma membrane sodium channels. For example, carbamazepine has been shown to modulate K_ATP_ channels^15,16^, GABA_A_ receptors^17^, and L-type Ca^2+^ channels^18^. While we have shown previously that carbamazepine acts on Nav1.7/*Scn9a* in beta-cells^4^, we cannot rule out additional effects on other ion channels that are relevant to beta-cell function and health without conducting additional experiments in future studies.

There are reports of carbamazepine effects that may be independent of electrical activity. Carbamazepine induces autophagy in hepatic and neuronal tissues, possibly reducing protein aggregation^19,20^. In mouse beta-cells, autophagy has been shown to maintain insulin secretion^21^, unfolded protein response machinery^22^, and cellular architecture^21,23^. Loss of autophagy induces hyperglycemia^21,23^ and is associated with decreased beta-cell mass^21^ and increased cell death^24^ in most, but not all, contexts^25^. Additional study is required to assess whether carbamazepine targets beta-cell autophagy.

Several epidemiological and clinical studies have linked the incidence of epilepsy to type 1 diabetes^26–29^, although it is unclear whether these diseases are linked by an underlying molecular mechanism. Topiramate, another sodium channel blocker and anti-epileptic drug, induced a prolonged period of euglycemia in a 43 year-old Caucasian patient concurrently treated with valproic acid for generalized seizures^30^. Topiramate, as well as other sodium channel blockers, have already been investigated for the treatment of neuropathic pain in type 2 diabetes^31^.

We were unable to resolve the role of the immune system in our model, at least with the assays we used and the time-points we studied, despite some prior evidence that carbamazepine can modulate the immune system^32–34^. Our data do not preclude the possibility that carbamazepine affects islet-specific immune cell activity locally, perhaps by affecting beta-cell immunogenicity. Future experiments analysing islet autoantigen specific T cells via tetramer staining could provide insight into the relative contribution of carbamazepine on islet specific cytotoxic T cell activity.

In summary, our studies suggest an anti-diabetic effect of carbamazepine that is robust in both *in vitro* and *in vivo* models of type 1 diabetes. Carbamazepine is used in the treatment of epilepsy^35^, but is also associated with side effects^36,37^. There is an unmet need for development of carbamazepine-like analogues as a potential new class of novel anti-diabetic drugs. Further work will focus on the elucidation of carbamazepine’s specific target(s) of action as well as the generation of blood-brain barrier impermeant derivatives to reduce unwanted side effects. If successful, this work may establish a new approach in preserving beta-cell function and/or survivability in type 1 diabetes.

## Methods

### Diabetes tracking and *in vivo* physiology

All animal procedures were approved by the University of British Columbia Animal Care Committee in accordance with the Canadian Council for Animal Care guidelines. Female, non-obese diabetic (NOD) mice housed in pathogen-free conditions were used for this study due to their higher incidence of spontaneous diabetes as compared to males. The oral carbamazepine dose we used was chosen based on a previous *in vivo* behavioural study^9^. Mice were weaned at 4–6 weeks of age onto irradiated 0.5% w/w carbamazepine (Sigma, C4024) supplemented LabDiet 5053 (5BTY) or control LabDiet 5053 formulated by TestDiet® in pellet form. A total of 42 mice were used to assess incidence of diabetes (‘incidence’ cohort) until 25 weeks of age. Mice were monitored weekly for body weight and 4-hour fasting blood glucose. Mice were considered diabetic at two consecutive measurements of fasting blood glucose above 16 mmol/L. Carbamazepine levels in serum from blood obtained by terminal cardiac puncture immediately after euthanization by CO_2_ were quantified after 1:1000 dilution (in assay diluent) using a commercial ELISA (Abraxis, 515585) according to the manufacturers protocol. An age matched, pre-diabetic (‘chase’ cohort) of mice (n = 5 for both control and carbamazepine treated) was euthanized 7 weeks of age (prior to the time when overt hyperglycemia is seen in any group). Glucose tolerance was assessed in the pre-diabetic ‘chase’ cohort of NOD mice (6 weeks of age) injected with 2 g/kg glucose. After resting for 1 week, a second injection of 2 g/kg glucose was given and 5 μl of blood was collected for insulin quantification by a commercial insulin ELISA kit (Alpco, 80-INSMS-E01).

### Histology, immunofluorescence and islet infiltration scoring

Pancreata were fixed in 10% formalin for 48 hours prior to storage in 70% ethanol at 4 °C. Paraffin embedded sections were prepared, sliced and stained with hematoxylin/eosin (H&E) or hematoxylin alone, with terminal deoxynucleotidyl transferase dUTP nick end labelling (TUNEL) by WaxIT Histology Services Inc. (Vancouver, BC). Whole H&E-stained slides were imaged by WaxIT Histology Services at 20X magnification. Five sections separated by 100 μm were scored blindly for infiltration of mononuclear cells into islets according to previous protocols^7^. TUNEL was imaged on a bright field Zeiss Axio Imager A1 microscope at 40X magnification, and the number of TUNEL-positive cells were normalized to the number of islets visible on each pancreatic section.

Three sections, separated by 100 μm, were deparaffinised, subjected to an antigen retrieval process, and stained according to a published protocol^38^ for quantification of insulin positive beta-cell area. Insulin was stained with 1:250 guinea pig anti-insulin (Abcam, ab7842) and 1:1000 goat anti-guinea pig AlexaFluor 594 (Invitrogen, A-11076). Slides were subsequently counter-stained with 4′, 6-diamidine-2′-phenylindole dihydrochloride (DAPI) in VECTASHIELD HardSet Antifade Mounting Medium (Vector Laboratories, H-1500) and whole slides were imaged using a 10X (NA 0.3) objective on an ImageXpress^MICRO^ robotic microscope and analyzed using MetaXpress 5.0 software (Molecular Devices). Beta-cell area was quantified by normalizing insulin positive area to the respective section size as determined by DAPI staining and was reported as a ratio of the two numbers. A subset of slides were also stained with 1:500 rabbit anti-PCNA (Cell Signaling Technologies, D3H8P) and 1:1000 goat anti-rabbit AlexaFluor 488 (Invitrogen, A-11008) and subsequently imaged on a Zeiss Axiovert 100 microscope using a 40X (NA 0.6) objective. Images were subsequently processed in Slidebook 5.5 (Intelligent Imaging Innovations) and Fiji (ImageJ) software^39^.

### Flow cytometry of pancreatic lymph nodes

Pancreatic lymph nodes were isolated from 7 week-old pre-diabetic NOD mice and single cell suspensions were obtained by passing them through a 40 μm cell strainer. Lymphocytes were stained with antibodies specific for: CD3 (conjugated with e450, used at 1:200, excited by a 405 nm laser)(48–0033), CD4 (conjugated with FITC, used at 1:100, excited by a 488 nm laser)(11–0042), and CD8 (conjugated with APC780, used at 1:100, excited by a 633 nm laser)(47–0081), all from eBioscience. 5000 live events were collected on a LSRII flow cytometer using BD FACSDiva software in the UBC Life Sciences Institute Flow Core and results were analyzed on Flowjo X 10.0.7 software.

### Multiplex cytokine assay

Mice were euthanized and cardiac puncture was performed to isolate whole blood upon diabetes development in the incidence cohort or at 7 weeks of age in the chase cohort. Blood was left to clot at room temperature for 15 minutes before centrifugation at 1000 times *g* at 4 °C. Samples were then stored at −20 °C until analysis. A panel of 7 cytokines from the chase cohort and a subset of the incidence cohort were assayed using the Bio-Plex Pro^TM^ Mouse Cytokine Th1 Panel (Bio-Rad, L6000004C6) according to the manufacturer’s instructions. Cytokine levels under the limit of threshold were assigned a value of zero when other analytes in the blood sample were detectable.

### Statistics

Unless indicated otherwise, all data are expressed as mean ± SEM. Statistical significance was defined at a threshold of p < 0.05. Diabetes incidence was measured by Kaplan-Meier analysis and quantified by log-rank test. Insulitis significance was quantified by Mann-Whitney U test with a minimum count of 100 islets quantified per treatment group. Due to a possible non-Gaussian distribution, beta-cell area was assessed for significance by both the Komolgorov-Smirnov and Mann Whitney U test. Unless otherwise noted, all other single variable based group comparisons were quantified by Student’s t-test using GraphPad Prism software.

### Data availability

All data generated during this study are included in this published article.
